# First detection and molecular characteristics of bopivirus from goats in China

**DOI:** 10.3389/fvets.2022.1033011

**Published:** 2022-12-01

**Authors:** Youwen Yang, Kehamo Abi, Yanmin Li, Chen Yang, Falong Yang

**Affiliations:** ^1^Department of Veterinary Medicine, College of Animal and Veterinary Sciences, Southwest Minzu University, Chengdu, China; ^2^Key Laboratory of Qinghai-Tibetan Plateau Animal Genetic Resource Reservation and Utilization, Chengdu, China

**Keywords:** bopivirus, molecular characteristics, goats, diarrhea, China

## Abstract

A metavirome analysis was performed and detected bopivirus in the diarrhoeal fecal samples of goats in China. A total of 136 fecal samples were collected from yeanlings between the dates of June 2021 and January 2022 in Sichuan province, China. Moreover, “Bopivirus B” strains were detected by a specific RT-PCR targeting the 3D gene of the virus. The results showed that the overall detection rate of “Bopivirus B” was 19.12% (26/136). Additionally, there was a higher detection rate (24.05%, 19/79) in the fecal samples collected from yeanlings with diarrhea compared to those from asymptomatic animals (12.28%, 7/57). In these samples, no other common diarrhea-causing pathogens were detected except for three enteric viruses, namely caprine enterovirus, caprine kobuvirus and caprine hunnivirus (with detection rates of 13.97, 13.97, and 8.82%, respectively). Subsequently, full-length VP4, VP2, VP3, and VP1 genes from “Bopivirus B”-positive samples were amplified, cloned, sequenced, and analyzed. The phylogenetic analysis performed on the VP1 genes revealed that the identified bopivirus belonged to genotype B1 (seven strains) and B2 (three strains) and presented a high genetic diversity. Furthermore, a complete genome sequence of a “Bopivirus B” strain (SWUN/B1/2022) was obtained using PCR from fecal sample of a diarrhoeal yeanling. The complete genome was 7,309 nucleotides in length with a standard picornavirus genome organization, and shares 93.10% and 91.10% nucleotide similarity with bopivirus B1 genotype strain ovine/TB14/2010-HUN and bopivirus B2 genotype strain goat/AGK16/2020-HUN, respectively. According to the species classification criteria put forward by the International Committee on Taxonomy of Viruses and VP1 genotype, the strain SWUN/B1/2022 belongs to the bopivirus B1. This strain has unique amino acid substitutions in the VP4, VP2, VP3, and VP1 genes. Moreover, genomic recombination analysis revealed that this strain may be a minor parental strain of bopivirus B1 ovine/TB14/2010-HUN. Evolutionary analysis based on the 2C and 3CD genes revealed that the new bopivirus B1 strain SWUN/B1/2022 presents a unique evolutionary pattern. This study provided evidence to suggest that “Bopivirus B” is circulating with substantial genetic diversity in goats in China at present, and the mixed infection of “Bopivirus B” with other enteric viruses should be considered to be a composite factor in the occurrence of viral diarrhea in goats.

## Introduction

Bopivirus is a newly recognized genus in the *Picornaviridae* family. Based on the current ICTV classification criteria, members of the same picornaviral genus should share >33% amino acid identity for capsid region P1 and >36% amino acid identity for non-structural proteins 2C and 3CD (1–3). At present, the genus *Bopivirus* contains one officially and two candidate recognized species, namely *Bopivirus A*, “Bopivirus B” and “bopivirus C” (www.picornaviridae.com). To date, members of the species *Bopivirus A* have been detected in cattle (4), whilst members of the species “Bopivirus B” have been identified in goats and sheep (5) and members of the species “Bopivirus C” in deer (3). Nonetheless, given that epidemiological data on bopivirus is limited and there is no standard cell culture system for isolating bopiviruses, the pathogenic potential of this virus in animals remains unknown. However, there are several members of *Picornaviridae* (including members of the genera *Enterovirus* and *Kobuvirus*) that are recognized as enteric viral pathogens, and these can cause diarrhea in ruminants (6). These findings suggest that bopivirus infection and its pathogenicity in ruminants are worthy of further investigation.

Furthermore, in 2021, members of the species “Bopivirus B” was only detected in fecal samples of sheep and goats in Hungary (5). Two complete “Bopivirus B” genomes (goat/AGK16/2020-HUN and ovine/TB14/2010-HUN) are available in the NCBI database. The genome of “Bopivirus B” is comprised of a 5' untranslated region (UTR), a large open reading frame (ORF), and a 3' UTR region, which encodes viral structural proteins P1 (VP1, VP4, VP3, and VP1), as well as non-structural proteins P2 (2A, 2B, and 2C) and P3 (3A, 3B, 3C, and 3D) (5).

China is one of the largest goat-raising countries in the world, yet there has been no information available regarding bopivirus in Chinese goats until now. During a viral genomic study examining diarrhea-causing viruses in goats, “Bopivirus B” sequences from the diarrhoeal fecal samples were identified, which indicates that “Bopivirus B” is present in Chinese goats. This may thus play a role in the occurrence of diarrhea in goats. The key aim of this study is to determine the prevalence of “Bopivirus B” in Chinese goats, as well as the molecular characteristics of the virus.

## Materials and methods

### Metagenomic sequencing of fecal samples in goats with diarrhea

In the Sichuan province of China in 2021, ten fecal samples of yeanlings (aged 2 d to 2 weeks) were collected from a goat farm experiencing an outbreak of diarrhea. A QIAamp Viral RNA Mini Kit (QIAGEN) was used to extract the nucleic acids from the fecal samples. In order to examine the viruses that may be involved in the development of diarrhea, the cDNA of 10 goat diarrhoeal fecal samples were placed in a pool for direct deep sequencing. The sequencing library was constructed according to the manufacturer's instructions (TruSeq RNA Sample Preparation Kit, USA). Subsequently, the nucleic acids were subjected to ultrasonic treatment to produce fragments (< 500 bp), and the DNA fragments were ligated through T4 DNA kinase and subsequently loaded on a HiSeq 4,000 (Illumina) for sequencing. Initially, the raw reads were processed using fastp (https://github.com/OpenGene/fastp) according to the following criteria: (i) trimmed reads shorter than 75 bp were discarded, (ii) a read was removed if N base is more than 5% of the read, (iii) the read was removed if more than 50% of the read had bases with quality lower than 15.

### Samples collection and nucleic acid extraction

Between June 2021 and January 2022, fecal samples were collected from 136 yeanlings at four goat farms in the Sichuan province of China. Of the 136 samples, 29 (20 diarrhoeal and nine non-diarrhoeal) were from yeanlings < 3 weeks old, 32 (22 diarrhoeal and 10 non-diarrhoeal) were 3–6 weeks old, and 75 (37 diarrhoeal and 38 non-diarrhoeal) were more than 6 weeks old (see Appendix A for sample detail). All of the samples were stored at −80°C. For viral RNA extraction, fecal samples were suspended in PBS (1:5) and centrifuged at 5,000 × g for 8 min, followed by treatment using a 0.22-μm filter. The total RNA was extracted from fecal suspension using Trizol reagent (Invitrogen) as described previously (7). After the centrifugation process was performed (4°C, 10 min, 12,000 × g), 200 μL of chloroform was added and the mixture was stirred thoroughly by inverting the tubes several times. The samples were centrifuged for 15 min at 12,000 × g and 450 μL uppermost aqueous layer was transferred into new Eppendorf tubes. RNA was precipitated for 10 min by adding 500 μL of isopropanol to the aqueous solution, after which it was centrifuged at 10,000 × g for 10 min, and washed with 75% ethanol. Finally, it was stored in 30 μL diethylpyrocarbonate (DEPC)-treated distilled water at −80°C. Viral RNA was reverse transcribed according to the manufacturer's instructions (TaKaRa, Japan), and the cDNA generated was stored at −20°C till use.

### Detection of “Bopivirus B” and common diarrhea-causing pathogens

A specific RT-PCR method developed in our laboratory was employed to detect members of the “Bopivirus B”. Briefly, primer sequences (299-F: 5′- TGACCTTGAACAGCGAGTTGAGAC-3′, 299-R: 5′- TCAAAATCATACACATAGGGAAAT-3′) was used to amplify a 299-bp 3D gene fragment (position 6,319–6,617 bp of the SWUN/B1/2022 complete genomic sequence, GenBank accession number ON044229). The amplification was performed in a 25 μL reaction volume containing 2 μL cDNA, 1 μL F/ R (0.05 μM), 12.5 μL of EmeraldAmp PCR Master Mix, and 8.5 μL RNase-free ddH_2_O. Moreover, RT-PCR or PCR were used to detect Peste des petits ruminants virus, bovine coronavirus, rotavirus A, bovine viral diarrhea virus, caprine kobuvirus, caprine enterovirus, *Escherichia coli* K99 and *Salmonella*, as described previously (8–14).

### Amplification of complete VP4, VP2, VP3, and VP1 genes from “Bopivirus B”-positive samples

In order to further understand the molecular characteristics of “Bopivirus B” in Chinese goats, “Bopivirus B”-positive samples were randomly selected from four goat farms (at least one sample from each farm), and complete VP4, VP2, VP3, and VP1 genes were amplified using RT-PCR with primers as shown in Table 1. All PCR products were purified using the Omega Gel kit, after which they were cloned into the pMD19-T simple vector (TaKaRa Bio Inc.) and sequenced by Sangon Biotech Co., Ltd. (Sangon Biotech Co., Ltd.).

**Table 1:** Primers for amplification of VP4, VP2, VP3 and VP1, and SWUN/B1/2022 genome.

**Primer names**	**Amplification size (bp)**	**Primer sequence (5'-3')**	**Amplified fragment**
Bo1F/Bo1R	750	TGATTCTTCCCTTCCCGCCAG	5'UTR and VP4 (partial)
		GATAGTGCTGCTGGTAGTAAT	
Bo2F/Bo2R	470	GGGCAGTCTACGCACTC	VP4 (partial) and VP2 (partial)
		TTCTGTGAGGTAGTCCAATCG	
Bo3F/Bo3R	1,060	CGATTGGACTACCTCACAGAA	VP2 (partial) and VP3 (partial)
		CACCCACCCGTCAACATCCGT	
Bo4F/Bo4R	798	CATCTCGCTCACACTTCTCTCT	VP3 (partial) and VP1 (partial)
		AGAGTGAAGGATTTTGGAAGCA	
Bo5F/Bo5R	853	ACCTCTGACCTGGGGCTGTGG	VP1 (partial), 2A (complete), and 2B (partial)
		AGAAGCCCACCCAATCACCGT	
Bo6F/Bo6R	1,964	GGTTCAGCGGGTAAGATTGC	2B (partial), 2C (complete), 3A (complete), 3B (complete), and 3C (partial)
		CCCGAAGGAAGAAGCCAGTG	
Bo7F/Bo7R	1,030	ATTGTGTGCCCGTCACCTTCC	3C (partial) and 3D (partial)
		CCATTGTCAGTGTAGGGAAGA	
Bo8F/Bo8R	1187	ATGACTATGCCGCCTCGCTCT	3D (partial) and 3'UTR
		CTAATTAATTCCAAATTCAGC	

### Amplification of complete genome of “Bopivirus B” SWUN/B1/2022

Eight pairs of primers (Table 1) were designed and used to amplify the genome sequence of the “Bopivirus B” strain SWUN/B1/2022. The sequence of 3' and 5' ends of the viral genome was also amplified using a Smart RACE cDNA amplification kit (TaKaRa Bio Inc.). The PCR products were cloned into the pMD19-T simple vector (TaKaRa Bio Inc.) for further sequencing (Sangon Biotech Co., Ltd., Shanghai, China).

### Sequence, phylogenetic, and recombination analysis

SOAP assembly software (DNASTAR Inc., WI, USA) was used to assemble the sequences. Meanwhile, the online ORF finder (https://www.ncbi.nlm.nih.gov/orffinder/) was employed to predict ORFs in the linear genomes. DNASTAR 7.0 software (DNASTAR Inc., WI, USA) was also used to analyze nucleotides and deduced aa sequence homologies. Sequence alignments and clustering were also performed by using ClustalW in MEGA 7.0 software. The tree was constructed using the neighbor-joining method with bootstrap values being calculated from 1,000 replications. The recombination events were evaluated using the SimPlot software (version 3.5.1) and the Recombination Detection Program RDP4.0 (version 4.9.5), which involved the RDP, MaxChi, GeneConv, Chimera, SiScan, 3Seq, and BootScan methods (15).

## Results

### High throughput sequencing

The sequence obtained by HiSeq 4000 was spliced *de novo* using SOAP software. According to nucleic acid BLAST searches, six sequence contigs had close similarity with bopivirus sequences (SRA accession number: SRR21808493), with lengths of 601, 621, 600, 657, 2,340, and 1,461 nt. The contigs were most similar to the bopivirus B1 genotype strain ovine/TB14/2010-HUN genome sequence (GenBank accession number: MW298057). These contigs corresponded to 699–1,299 bp; 1,300–1,920 bp; 2,112–2,711 bp; 2,662–3,327 bp; 3,328–5,666 bp, and 5,668–7,308 bp of the genome sequence of strain ovine/TB14/2010-HUN.

### Detection of “Bopivirus B” in goat feces

“Bopivirus B” was detected in goats from all four regions, with an overall infection rate of 19.12% (26/136) (varying between 14.29 and 30.00% in different regions, see Table 2 for more information). Further analysis showed that 45.0/11.1%, 27.27/10.0%, and 10.81/13.16% of the “Bopivirus B”-positive diarrhoeal/non-diarrhoeal samples were from goats aged < 3 weeks, 3–6 weeks, and more than 6 weeks, respectively (Table 3). Moreover, the samples were also screened for nine other common diarrhea-causing pathogens, with the results showing that three enteric viruses were present, namely caprine enterovirus, caprine kobuvirus and caprine hunnivirus. These three pathogens were detected with infection rates of 13.97, 13.97, and 8.82%, respectively. No other common diarrhea-causing pathogens (e.g., peste des petits ruminants virus, bovine coronavirus, rotavirus A, bovine viral diarrhea virus, *Escherichia coli* K99 and *Salmonella* spp.) could be found in the samples. These results demonstrated that young animals are more susceptible to “Bopivirus B”, as well as caprine enterovirus, caprine kobuvirus and caprine hunnivirus. Moreover, these viruses are related to the occurrence of diarrhea in yeanlings.

**Table 2:** Overall infection of “Bopivirus B” in Chinese goats.

**Regions**	**Farms**	**Samples**	**Infection rates in diarrhea goats**	**Infection rates in non-diarrhea goats**	**Total**
Zigong	1	56	17.50%	6.25%	14.29%
Jintang	1	33	27.27%	9.09%	21.21%
Shuangliu	1	20	33.33%	20.00%	30.00%
Xinjin	1	27	50.00%	16.00%	18.52%
Total	4	136	24.05%(19/79)	12.28%(7/57)	19.12%

**Table 3:** Infection of “Bopivirus B” and other enteric viruses in goats of different ages.

**Viruses**	**Infection rates (diarrhoeal/non-diarrhoeal) (*****n*** = **136, 79/57)**			**Total**
	** < 3 weeks (*n* = 29)**	**3–6 weeks (*n* = 32)**	**>6 weeks (*n* = 75)**	
“Bopivirus B”	45.00/11.11%	27.27/10.00%	10.81/13.16%	19.12%
Caprine entervirus	20.00/11.11%	13.64/10.00%	18.92/7.89%	13.97%
Caprine kobuvirus	20.00/11.11%	13.64/0.00%	21.62/7.89%	13.97%
Caprine hunnivirus	20.00/0.00%	22.73/0.00%	5.41/2.63%	8.82%

### Molecular characterization of the VP4, VP2, VP3, and VP1 genes of the “Bopivirus B”

To characterize the “Bopivirus B” strains detected in the goats, complete VP4, VP2, VP3, and VP1 genes were simultaneously amplified, cloned and sequenced from the “Bopivirus B”-positive samples (this was performed on at least one sample from each of four farms). From the nine “Bopivirus B”-positive samples, the complete VP4 gene was cloned. All nine of the VP4 genes were 255-bp in length and encoded 85 aa residues (GenBank accession number: ON044231- ON044238). The nine VP4 genes shared 91.4–100% nt similarity and 97.6–100% aa similarity. Moreover, they shared 91.8–94.5% nt similarity and 97.6–100% aa similarity with the bopivirus B1 strain ovine/TB14/2010-HUN VP4 gene. They also shared 92.5–95.7% nt similarity and 97.6–100% aa similarity with the bopivirus B2 strain goat/AGK16/2020-HUN VP4 gene. Further analysis revealed that five of nine VP4 genes shared one aa substitution (D68E) in the VP4 protein, in comparison to the sequence of bopivirus B1 ovine/TB14/2010-HUN. Furthermore, seven of the nine VP4 genes shared one aa substitution (H44S) in the VP4 protein, in comparison to the bopivirus B2 strain goat/AGK16/2020-HUN.

The complete VP2 gene was cloned from the nine bopivirus-positive samples. All of the nine VP2 genes were 714-bp in length and encoded 238 aa residues (GenBank accession number: ON044231- ON044238). The nine VP2 genes shared 89.2–100% nt similarity and 97.9–100% aa similarity. They also shared 88.8–91.6% nt similarity and 97.1–97.9% aa similarity with the bopivirus B1 strain ovine/TB14/2010-HUN VP0 gene. Additionally, they shared 89.9–90.5% nt similarity and 97.1–99.2% aa similarity with the bopivirus B2 strain goat/AGK16/2020-HUN VP2 gene. Moreover, further analysis revealed that the nine VP2 genes shared one aa substitution (F142T) in the VP2 protein, and four of these nine VP2 genes shared four identical aa substitutions (S135G, E145Q, Q137T, and D150N) in the region of the VP2 gene, in comparison to the sequence of bopivirus B1 ovine/TB14/2010-HUN. Additionally, there were four identical aa substitutions (K39R, T42A, D135S, G, and S210T) in the VP2 protein compared with the bopivirus B2 strain goat/AGK16/2020-HUN.

From the nine “Bopivirus B”-positive samples, complete VP3 genes were cloned. All nine of the VP3 genes were 678 bp in length and encoded 226 aa residues (GenBank accession number: ON044231- ON044238). The nine VP3 genes shared 90.7–99.7% nt similarity and 97.8–100% aa similarity, as well as 91.6–92.8% nt similarity, 97.8–98.7% aa similarity with the bopivirus B1 strain ovine/TB14/2010-HUN VP3 gene, and 90.3–91.4% nt similarity and 98.2–98.7% aa similarity with the bopivirus B2 strain goat/AGK16/2020-HUN VP3 gene. Additionally, it was revealed in a further analysis that nine VP3 genes shared two identical aa substitutions (E56R, K, K57D, N) in the VP3 protein, in comparison to bopivirus B1 strain ovine/TB14/2010-HUN. The nine VP3 genes shared two identical aa substitutions (SA95A, V200A) in the VP3 protein, in comparison to bopivirus B2 strain goat/AGK16/2020-HUN.

**Figure 1:**
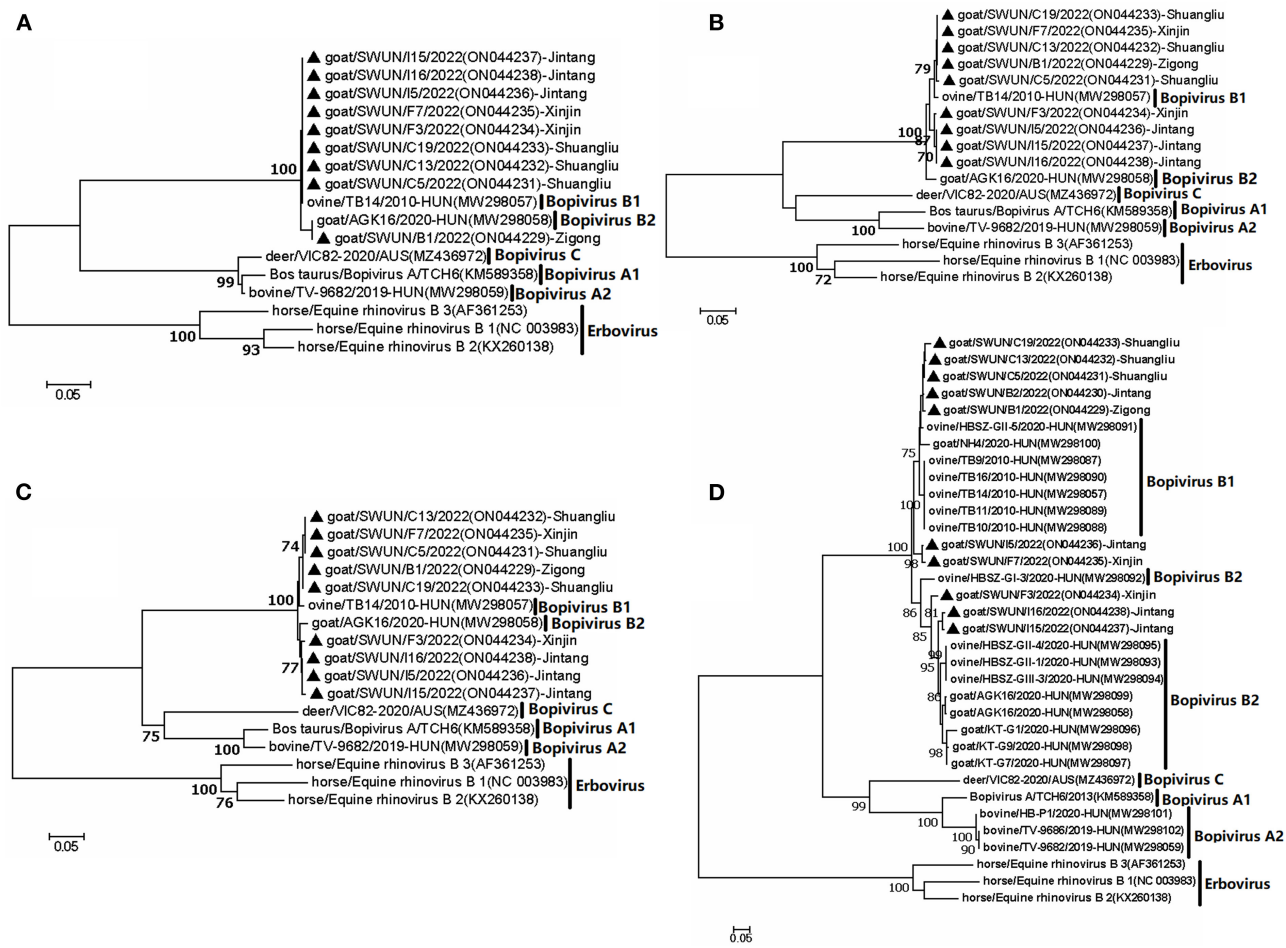
Phylogenetic relationship of bopiviruses from this study with representative members from genus *Bopivirus* based on aa similarity of the complete VP4 **(A)**, VP2 **(B)**, VP3 **(C)**, and VP1 **(D)**. The trees were constructed using the neighbor-joining method in MEGA 7.0 program. The bootstrap values were calculated from 1,000 replications.The black triangles indicate that the corresponding sequences were from the strains in this study.

Furthermore, a phylogenetic tree was constructed based on VP4, VP2 and VP3 genes from the nine “Bopivirus B” strains in the present study, as well as genes from other species of *Bopivirus* in the NCBI database. The findings revealed that these nine strains clustered with the bopivirus B1 strain ovine/TB14/2010-HUN and the bopivirus B2 strain goat/AGK16/2020-HUN on a large independent branch (Figures 1A–C).

From the ten “Bopivirus B”-positive samples, complete VP1 genes were cloned. All ten of the VP1 genes were 933 bp in length and encoded 311 aa residues (GenBank accession number: ON044229- ON044238). The ten VP1 genes shared 75.9–98.8% nt similarity and 83–99% aa similarity, as well as 76–90.5% nt similarity, 72–95.8% aa similarity with the bopivirus B1 strain ovine/TB14/2010-HUN VP1 gene, and 74.5–90.9% nt similarity, 80.7–96.8% aa similarity with the bopivirus B2 strain goat/AGK16/2020-HUN VP1 gene.

Because VP1 is often employed in genotyping (16, 17), and thus the potential genotype of bopivirus VP1 were subjected to further analysis. Pairwise distances (p-distances) between the seven VP1 nucleotide sequences (SWUN/B12022, SWUN/B2/2022, SWUN/C13/2022, SWUN/C19/2022, SWUNC5/2022, SWUN/F7/2022, SWUN/I5/2022) in the present study and bopivirus B1 reference sequence was 0.095–0.138, and p-distances between the remaining three VP1 nucleotide sequences (SWUN/F3/2022, SWUN/I15/2022, and SWUN/I16/2022) and bopivirus B2 reference sequence was 0.092–0.147. Moreover, prior research has shown that there is a p-distance of < 0.16 between the bopivirus intragenotype (5). These findings indicated that the seven VP1 genes belong to bopivirus B1, while the remaining 3 VP1 genes from this study belong to bopivirus B2.

A phylogenetic tree was created using all complete VP1 genes from this study and the VP1 genes of other species of *Bopivirus* in the GenBank and this tree showed that all of the bopivirus B VP1 sequences fell into five distinct branches. Interestingly, two of the seven bopivirus B1 VP1 sequences identified in this study were clustered into a small independent branch and this differed from the known bopivirus B1 VP1 sequences. Meanwhile, the remaining five of seven bopivirus B1 VP1 sequences could be clustered into a small independent branch. When combined all bopivirus B1 strains, they could be clustered into a large independent branch. Moreover, the three bopivirus B2 VP1 genes identified in the present study could be clustered into two small independent branches, and, combined with all bopivirus B2 strains, they could be clustered into a large independent branch (Figure 1D).

**Figure 2:**
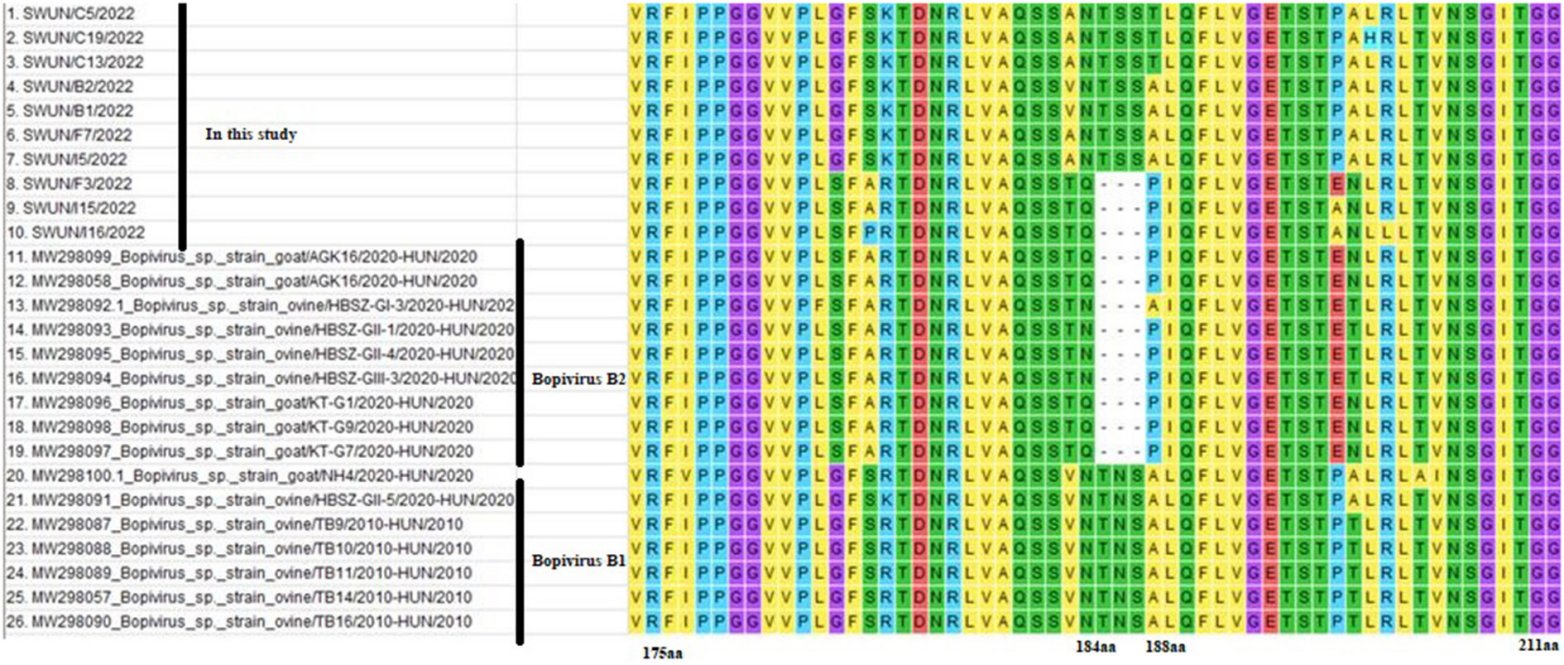
Amino acid variants of VP1 protein from different “Bopivirus B” strains. Clustal W was used to performed sequence alignments in MEGA 7.0 software.

Meanwhile, a further analysis revealed that ten VP1 genes shared five identical aa substitutions (I116L, S131A, T, G, T199A, N, T254V, E, and A302E, T) in the VP1 protein, compared with bopivirus B1 strain ovine/TB14/2010-HUN. Ten VP1 genes shared seven identical aa substitutions (I36V, A86D, Q120S, S132G, Q, L, V298T, A, A302E, T, and T309S, A) in the VP1 protein, in comparison to the bopivirus B2 strain goat/AGK16/2020-HUN. Additionally, all of the bopivirus B2 genotype strains have three aa deletions between N184 and A188, compared with other “Bopivirus B” genotype strains (Figure 2).

### Full genomic sequence of the strain SWUN/B1/2022

**Figure 3:**
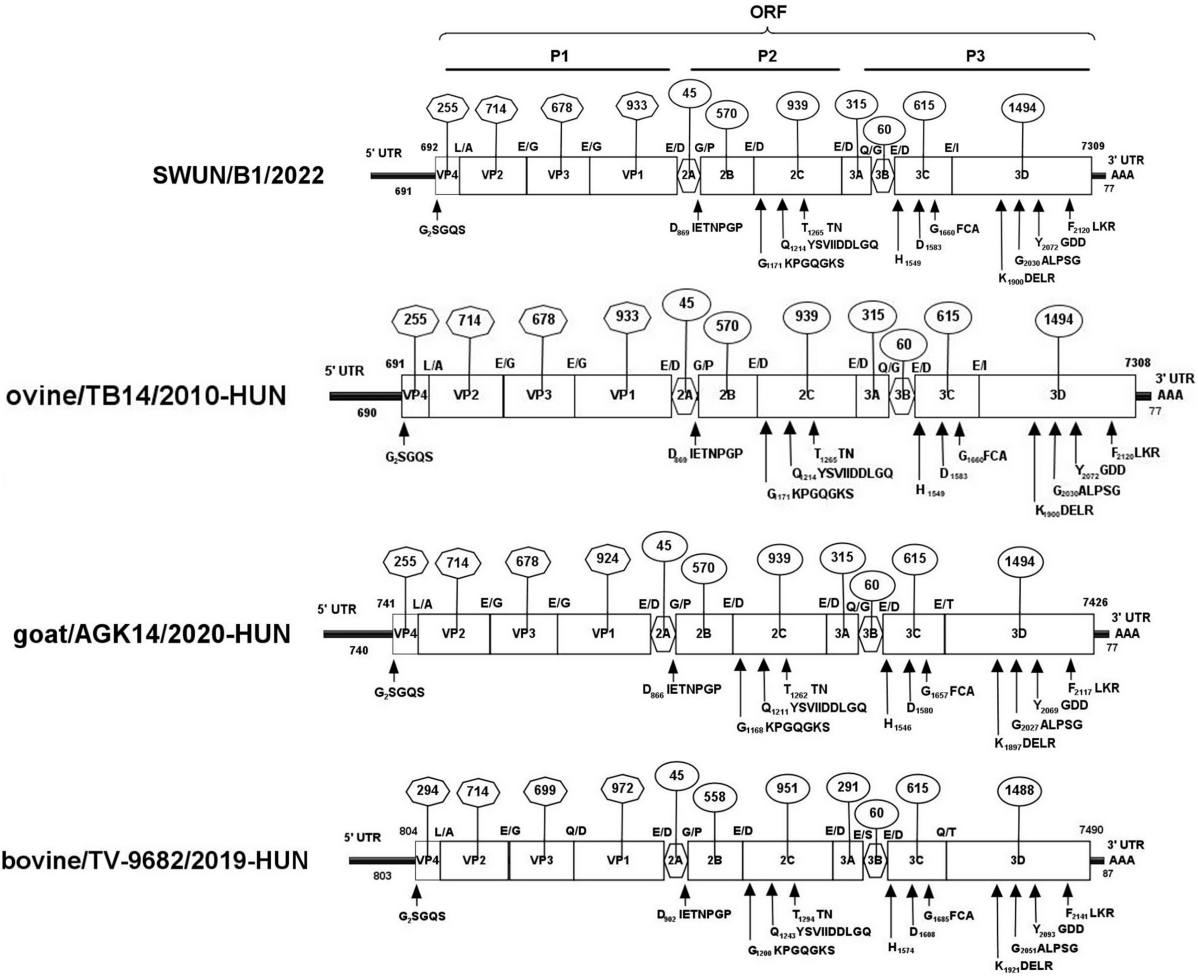
Genomic organization of the “Bopivirus B” strain SWUN/B1/2022 identified in this study and the other reference bopivirus strains in the GenBank database. Circled numbers indicate the length of each gene, and plain numbers indicate the location of the genome. The positions and sequences of conserved amino acid motifs of 2C helicase, 3C protease, and 3D^RdRp^ (RNA-dependent RNA polymerase) are shown under each map of the viruses.

Overlapping fragments covering the entire genome were amplified and cloned separately from a diarrhoeal fecal sample from a young goat using RACE and RT-PCR. The assembled genome sequence of the strain SWUN/B1/2022 was 7,309 nt in length. It contains a 691 nt 5′UTR and a 77 nt 3′UTR with a 6,618-nt ORF that encodes a polyprotein, P1 (VP4, VP2, VP3, and VP1), and non-structural proteins P2 (2A-2C) and P3 (3A-3D) (Figure 3).

The genome sequences of the strain SWUN/B1/2022 and those of the seven representative bopivirus species available in the NCBI database were compared and the findings revealed that this bopivirus was most similar to the bopivirus B1 strain ovine/TB14/2010-HUN, with 93.10% nt similarity. This bopivirus shared 91.10% nt similarity with bopivirus B2 strain goat/AGK16/2020-HUN and shared 60.90–61.50% nt similarity with other bopivirus sequences. The SWUN/B1/2022 strain had the highest aa similarity with bopivirus B1 strain ovine/TB14/2010-HUN, with similarity of 97.3% (complete polyprotein), 97.7% (P1), 99% (2C), and 95.1% (3CD) (Table 4). These findings indicate that the bopivirus strain belongs to “Bopivirus B”.

**Table 4:** Nucleotide and deduced amino acid similarity of the new “Bopivirus B” strain SWUN/B1/2022 and other five representative species of *Bopivirus* in the NCBI database.

**Strains (genotype/host)**	**GenBank accession number**	**Genome nucleotide identities**	**Amino acid identities**				
			**Complete polyprotein**	**P1**	**2C**	**3CD**	**3D**
TCH6 (A1/cattle)	KM589358	60.90%	60.20%	58.60%	65.80%	66.30%	67.70%
TV-9682/2019 (A2/cattle)	MW298059.1	61.10%	60.10%	57.90%	65.80%	67.10%	68.60%
VIC82-2020/AUS (C/deer)	MZ436972	61.50%	60.50%	58.70%	63.30%	68.50%	69.60%
TB14/2010 (B1/sheep)	MW298057.1	93.10%	97.30%	97.70%	99.00%	95.10%	97.40%
AGK16/2020 (B2/goat)	MW298058.1	91.10%	95.50%	92.90%	99.00%	94.80%	97.40%

**Figure 4:**
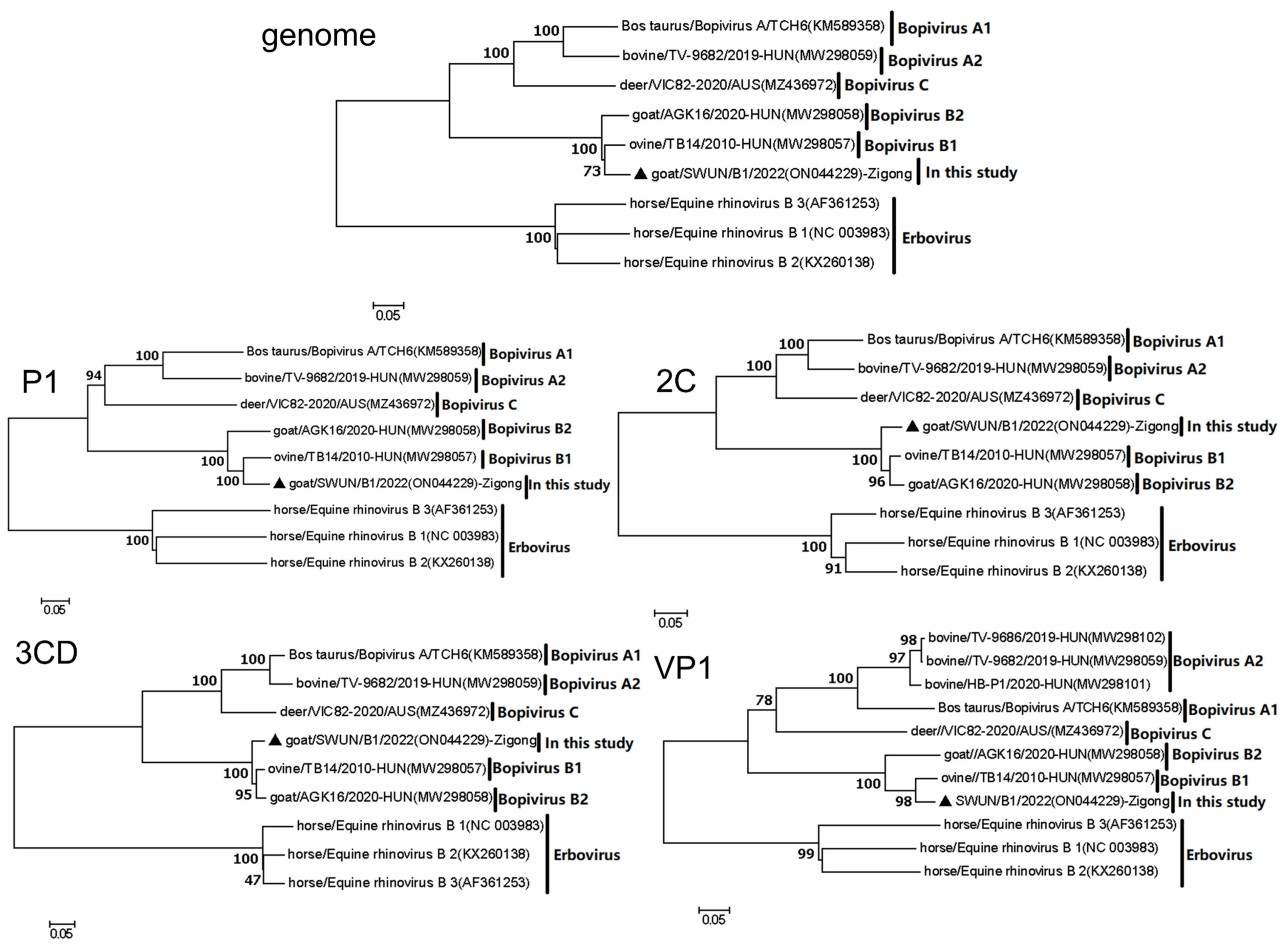
Phylogenetic relationship of bopiviruses from this study with representative members from genus *Bopivirus* based on nt similarity of the P1, 2C, 3CD, VP1, and complete genome. The trees were constructed using the neighbor-joining method in MEGA 7.0 program. The bootstrap values were calculated from 1,000 replications. The black triangles indicate that the corresponding sequences were from the strains in this study.

Evolutionary tree analysis revealed that the strain SWUN/B1/2022, together with bopivirus B1 ovine/TB14/2010-HUN, clustered on a small independent branch based on nucleotides of the genome, P1 and VP1. However, the bopivirus B1 SWUN/B1/2022 clustered into an independent branch based on nucleotides of the 2C and 3CD genes. These results indicate that this may be a new type of “Bopivirus B” strain (Figure 4).

In comparison to the bopivirus B1 strain ovine/TB14/2010-HUN, the new “Bopivirus B” strain SWUN/B1/2022 has two aa substitutions (S44H and D68E) in the VP4 protein, two aa substitutions (E141A and F142T) in the VP2 protein, and three aa substitutions (E56K, K57N, and Y61F) in the VP3 protein, and 12 aa substitutions (A102S, I116L, E130G, S131T, L133S, S134A, R137K, N185S, T199A, S248N, T254V, and A298T) in the VP1 protein. When compared with bopivirus B2 strain goat/AGK16/2020-HUN, the strain SWUN/B1/2022 has two aa substitutions (E141A and F142T) in the VP2 protein and four aa substitutions (A55D, D57N, S195A, and V200A) in the VP3 protein and 47 aa substitutions in the VP1 protein.

### Genomic recombination analysis

**Figure 5:**
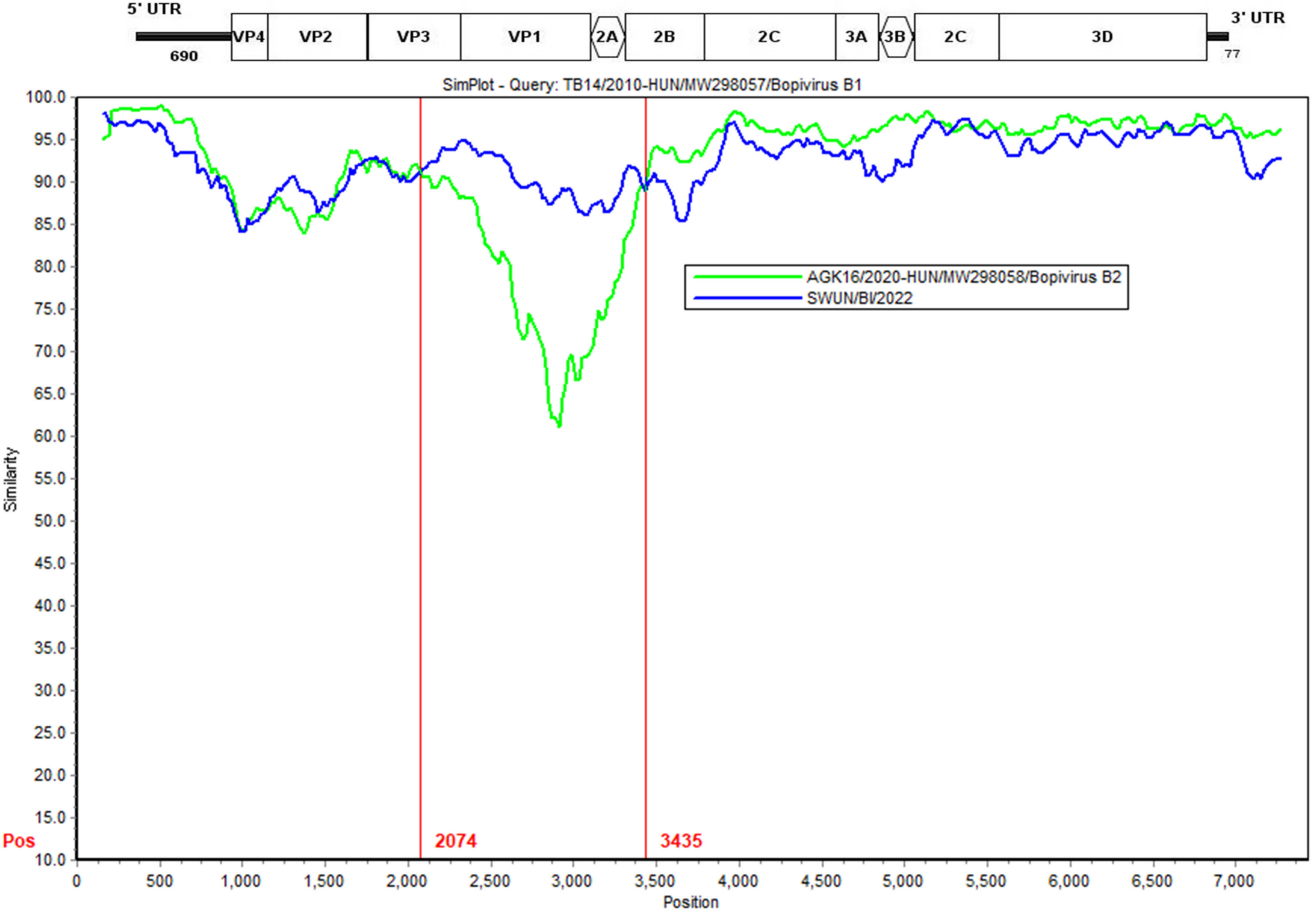
Recombination analysis of the bopivirus strains using SimPlot 3.5.1. The bopivirus B1 strain ovine/TB14/2010-HUN (GenBank accession number MW298057) and the bopivirus B2 strain goat/AGK16/2020-HUN (GenBank accession number MW298058) are shown. The potential recombination region is located at 2,074–3,435 bp of the bopivirus genome.

Further analysis findings suggest that there was a recombination event in the bopivirus B1 strain ovine/TB14/2010-HUN based on SimPlot 3.5.1 and RDP 4.0 (seven methods, recombinant score: 0.439). In the complete-length genome sequence, the recombination breakpoint was estimated being situated on the VP3-2B region at position 2,074–3,435 bp. This highlighted that the putative major parental strain was a form of bopivirus B2 goat/AGK16/2020-HUN (GenBank accession number: MW298058) and this identified a potential parental strain as the new “Bopivirus B” strain SWUN/B1/2022 in this study (GenBank accession number: ON044229) (Figure 5).

## Discussion

### Prevalence of the “Bopivirus B” from goats in China

In the present work, it was revealed that 19.12% (26/136) of goat fecal samples were tested positive for “Bopivirus B” by specific RT-PCR. Moreover, the distribution of the “Bopivirus B” spread across four goat farms, with the two most distant farms being located more than 190 km apart. This indicates that “Bopivirus B” is widely circulating in China. Further analysis showed “Bopivirus B” was high prevalent in young goats, whilst the caprine enterovirus, caprine kobuvirus and caprine hunnivirus were also found to be present in the goat diarrheic samples. This indicates that the mixed infection of “Bopivirus B” with other enteric viruses should be considered to be a composite factor for the development of viral diarrhea in goats in China. Nonetheless, further investigations are required to determine the relationship between “Bopivirus B” infection and the capacity of “Bopivirus B” to cause diarrhea in goats (particularly young goats).

### Goat strain SWUN/B1/2022 may represent a new member of “Bopivirus B”

The complete genome of bopivirus strain SWUN/B1/2022 was obtained from a fecal sample of a diarrhoeal goat. The complete genomic sequence was 7,309 nt in length, with a typical genome organization of picornaviruses, and contains a 6,618 nt ORF that encodes a polyprotein of 2,206 aa. The results revealed that the strain SWUN/B1/2022 is most closely related to the bopivirus B1 strain ovine/TB14/2010-HUN with 93.10% nt similarity in the genome and over 90% aa similarity for the polyproteins, P1, 2C, and 3CD (Table 4). Using the ICTV species classification criteria for bopivirus, the new bopivirus identified in this study has been classified as a “Bopivirus B” member (1, 2). The strain SWUN/B1/2022 has three significant characteristics, namely (i) This strain may be a minor parental strain of bopivirus B1 ovine/TB14/2010-HUN caused during a recombination event in the VP3-2B regions; (ii) it has unique amino acid substitutions in the VP4, VP2, VP3, and VP1 genes; (iii) there was a unique evolutionary pattern in the nucleotides of the 2C and 3CD genes. These results indicate that the strain may be a new “Bopivirus B” member.

Moreover, it has been reported that recombination events have occurred in picornaviruses (which are located in the polyprotein, VP1, 2C and VP3 genes) (18–22). These recombination events play a critical role in the evolution of picornaviruses and can result in the development of new genotypes, as well as changes in the host range (19–21, 23, 24). Nevertheless, recombination events in bopivirus have not been studied. In the present work, the bopivirus B1 strain ovine/TB14/2010-HUN was identified as a recombinant strain following an event in the VP3-2B gene. In this case, the bopivirus B2 goat/AGK16/2020-HUN served as the putative major parental strain and the new bopivirus B strain SWUN/B1/2022 as the possible minor parental strain. This indicates that the bopivirus B1 strain ovine/TB14/2010-HUN may be derived from natural recombination of bopivirus B2 strain goat/AGK16/2020-HUN and the strain SWUN/B1/2022. These findings provide a reference for understanding the evolution of “Bopivirus B”.

### Molecular characterization of VP4, VP2, and VP1 gene of “Bopivirus B”

VP2 and VP4 in picornaviruses may be involved in cellular receptor recognition, antigenic diversity, and viral pathogenesis (25–27). Additionally, picornavirus VP1 may play a role in cellular receptor recognition and viral pathogenesis (28, 29). Interestingly, in comparison to the bopivirus B1 strain ovine/TB14/2010-HUN, five of nine VP4 genes from this study shared one aa substitution (D68E) in the VP4 protein, nine VP2 genes shared one aa substitution (F142T) in the VP2 protein, and ten VP1 genes shared five identical aa substitutions in the VP1 protein. Moreover, in comparison to the bopivirus B2 strain goat/AGK16/2020-HUN, seven of nine VP4 genes shared one aa substitution (H44S) in the VP4 protein, and four of these nine VP2 genes shared four identical aa substitutions (S135G, E145Q, Q137T, and D150N) in the VP2 genes. Meanwhile, ten VP1 genes shared seven identical aa substitutions in the VP1 protein. Nonetheless, there is very little evidence to determine whether these unique aa mutations impact the function of VP4, VP2, and VP1. Thus, further investigations are required.

VP1 has been used for genotyping of picornaviruses (30–32). Two genotypes (bopivirus B1 and B2) have been reported in goats and sheep (5). Interestingly, the p-distances between the seven VP1 nucleotide sequences from this study and bopivirus B1 reference sequence, and the p-distances between the remaining three VP1 nucleotide sequences and bopivirus B2 reference sequence, are below 0.16. Based on the VP1 genotype classification assay (5), the seven VP1 sequences discussed above have been identified as bopivirus B1. Furthermore, the three VP1 genes mentioned above have been identified as bopivirus B2. Further analysis has revealed that two of seven VP1 bopivirus B1 genes Identified in this study clustered into a small independent branch, with the remaining five bopivirus B1 VP1 genes clustering with known bopivirus B1 VP1 genes on a large independent branch. Moreover, the three bopivirus B2 VP1 in this work can be clustered into two small independent branches. They also clustered with the bopivirus B2 strains on a large independent branch. These findings indicated that “Bopivirus B” VP1 protein has significant genetic diversity. However, given the limited sequences of bopivirus VP1 available in public database, the effects of different VP1 lineages on its antigenicity, host and tissue tropism are unknown at present, meaning that further studies are needed to establish such association.

In conclusion, this study is the first we are aware of to report prevalence and molecular characteristics of “Bopivirus B” in Chinese goats. Further studies are required to investigate the possible role of “Bopivirus B” as a diarrhea-causing pathogen in goats and to further clarify the epidemiological significance of these findings.

## Data availability statement

The datasets presented in this study can be found in online repositories. The names of the repository/repositories and accession number(s) can be found below: https://www.ncbi.nlm.nih.gov/, ON044229-ON044238.

## Ethics statement

The studies involving animals were reviewed and approved by Southwest Minzu University Institutional Animal Care and Use Committee. Written informed consent was obtained from the owners for the participation of their animals in this study, and written informed consent was obtained from the owners of the animals for the publication of any potentially identifiable images or data included in this article.

## Author contributions

FY and KA conceptualized and designed the experiments. YY and CY collected the samples and performed the experiments. FY, YY, KA, and CY collected samples and analyzed the data. FY, KA, and YL wrote and revised the manuscript. All authors contributed to the article and approved the submitted version.

## Funding

This work was funded by Sichuan provincial Key R&D projects (2021YFN0008) and Key Laboratory of Veterinary Medicine of Universities of Sichuan Province (2021PTJS34).

## Conflict of interest

The authors declare that the research was conducted in the absence of any commercial or financial relationships that could be construed as a potential conflict of interest.

## Publisher's note

All claims expressed in this article are solely those of the authors and do not necessarily represent those of their affiliated organizations, or those of the publisher, the editors and the reviewers. Any product that may be evaluated in this article, or claim that may be made by its manufacturer, is not guaranteed or endorsed by the publisher.
