# Supplementary material for: First detection and molecular characteristics of bopivirus from goats in China
Source: Front Vet Sci. 2022 Dec 1;9:1033011. doi: 10.3389/fvets.2022.1033011 (PMC9753977; doi:10.3389/fvets.2022.1033011)
Supplement: Supplementary file 1 [file Data_Sheet_1.DOCX]

Supplementary Material

1. **Appendix Table A**

Detailed background information of individual samples used for the epidemiological investigations as well as the results of bopivirus B 3D screening and three enteric viruses RT-PCR reactions. ID: identification marks. Age group I: < 3 weeks, Age group II: 3 to 6 weeks, Age group III: >6 weeks. AS: asymptomatic, DI: diarrhoea. Bopivirus-3D-RT- PCR positive samples are marked with bold.

| Farm location (ID) | Sample ID | Host | Age group | Health status | Bopivirus B-PCR | other infectious viruses |
| --- | --- | --- | --- | --- | --- | --- |
| Zigong | B1 | ovine | III | DI | - | hunnivirus |
| Zigong | B2 | ovine | II | DI | - |  |
| Zigong | B3 | ovine | II | DI | - | hunnivirus |
| Zigong | B4 | ovine | I | DI | - | hunnivirus、kobuvirus |
| Zigong | B5 | ovine | III | DI | **+** | kobuvirus |
| Zigong | B6 | ovine | III | DI | - |  |
| Zigong | B7 | ovine | II | DI | - | enterovirus、hunnivirus、kobuvirus |
| Zigong | B8 | ovine | I | DI | - | enterovirus、 hunnivirus |
| Zigong | B9 | ovine | III | DI | - | enterovirus、kobuvirus |
| Zigong | B10 | ovine | I | DI | **+** | enterovirus |
| Zigong | B11 | Ovine | I | DI | - | enterovirus、hunnivirus、kobuvirus |
| Zigong | B12 | Ovine | III | DI | - | enterovirus、kobuvirus |
| Zigong | B13 | ovine | III | DI | **+** | kobuvirus |
| Zigong | B14 | ovine | II | DI | - |  |
| Zigong | B15 | ovine | II | DI | - |  |
| Zigong | B16 | ovine | III | DI | - |  |
| Zigong | B17 | ovine | I | DI | - |  |
| Zigong | B18 | ovine | I | DI | - |  |
| Zigong | B19 | ovine | II | DI | - |  |
| Zigong | B20 | ovine | III | DI | - |  |
| Zigong | B21 | ovine | III | DI | - |  |
| Zigong | B22 | ovine | III | DI | - | enterovirus |
| Zigong | B23 | ovine | III | DI | - | enterovirus |
| Zigong | B24 | ovine | II | DI | **+** |  |
| Zigong | D1 | ovine | III | AS | - |  |
| Zigong | D2 | ovine | I | AS | - |  |
| Zigong | D3 | ovine | I | AS | - |  |
| Zigong | D4 | ovine | III | AS | - |  |
| Zigong | D5 | ovine | II | AS | - |  |
| Zigong | D6 | ovine | III | DI | - | hunnivirus |
| Zigong | D7 | ovine | III | DI | - |  |
| Zigong | D8 | ovine | III | AS | - |  |
| Zigong | D9 | ovine | I | DI | - |  |
| Zigong | D10 | ovine | I | DI | **+** |  |
| Zigong | D11 | ovine | II | AS | - |  |
| Zigong | D12 | ovine | II | AS | - |  |
| Zigong | D13 | ovine | I | AS | - |  |
| Zigong | D14 | ovine | III | AS | - |  |
| Zigong | D15 | ovine | III | AS | - |  |
| Zigong | D16 | ovine | III | AS | - |  |
| Zigong | D17 | ovine | III | AS | - |  |
| Zigong | D18 | ovine | III | DI | - | kobuvirus |
| Zigong | D19 | ovine | II | DI | **+** | kobuvirus |
| Zigong | D20 | ovine | III | AS | **+** | hunnivirus |
| Zigong | D21 | ovine | I | DI | **+** | hunnivirus |
| Zigong | G1 | ovine | III | AS | - |  |
| Zigong | G2 | ovine | III | DI | - |  |
| Zigong | G3 | ovine | III | DI | - |  |
| Zigong | G4 | ovine | II | DI | - |  |
| Zigong | G5 | ovine | III | DI | - |  |
| Zigong | G6 | ovine | III | DI | - | enterovirus |
| Zigong | G7 | ovine | III | DI | - |  |
| Zigong | G8 | ovine | I | AS | - |  |
| Zigong | G9 | ovine | I | DI | - |  |
| Zigong | G10 | ovine | III | DI | - |  |
| Zigong | G11 | ovine | III | DI | - |  |
| Jintang | H1 | ovine | III | AS | - |  |
| Jintang | H2 | ovine | III | AS | - |  |
| Jintang | H3 | ovine | III | DI | - | kobuvirus |
| Jintang | H4 | ovine | I | DI | - |  |
| Jintang | H5 | ovine | III | DI | - | enterovirus |
| Jintang | H6 | ovine | I | DI | - | kobuvirus |
| Jintang | H7 | ovine | III | DI | - |  |
| Jintang | H8 | ovine | III | DI | - |  |
| Jintang | I1 | ovine | I | DI | - |  |
| Jintang | I2 | ovine | II | DI | - |  |
| Jintang | I3 | ovine | I | AS | - |  |
| Jintang | I4 | ovine | III | DI | - |  |
| Jintang | I5 | ovine | II | DI | **+** | hunnivirus |
| Jintang | I6 | ovine | I | AS | **+** |  |
| Jintang | I7 | ovine | III | AS | - |  |
| Jintang | I8 | ovine | I | AS | - |  |
| Jintang | I9 | ovine | I | DI | **+** |  |
| Jintang | I10 | ovine | II | DI | - | hunnivirus |
| Jintang | I11 | ovine | II | DI | - |  |
| Jintang | I12 | ovine | II | DI | - | hunnivirus |
| Jintang | I13 | ovine | II | DI | - |  |
| Jintang | I14 | ovine | II | DI | **+** |  |
| Jintang | I15 | ovine | II | DI | **+** | enterovirus |
| Jintang | I16 | ovine | II | DI | **+** |  |
| Jintang | I17 | ovine | III | DI | **+** |  |
| Jintang | I18 | ovine | III | AS | - |  |
| Jintang | I19 | ovine | II | AS | - |  |
| Jintang | I20 | ovine | III | AS | - |  |
| Jintang | I21 | ovine | II | AS | - |  |
| Jintang | I22 | ovine | III | DI | - |  |
| Jintang | I23 | ovine | III | DI | - |  |
| Jintang | I24 | ovine | III | DI | - | enterovirus |
| Jintang | I25 | ovine | II | AS | - |  |
| Shuangliu | C1 | ovine | III | DI | - | kobuvirus |
| Shuangliu | C2 | ovine | I | DI | **+** | enterovirus |
| Shuangliu | C3 | ovine | III | DI | - | kobuvirus |
| Shuangliu | C4 | ovine | III | AS | - |  |
| Shuangliu | C5 | ovine | II | DI | - |  |
| Shuangliu | C6 | ovine | II | DI | - |  |
| Shuangliu | C7 | ovine | III | DI | **+** |  |
| Shuangliu | C8 | ovine | II | DI | - | kobuvirus |
| Shuangliu | C9 | ovine | II | DI | - | enterovirus |
| Shuangliu | C10 | ovine | II | AS | - |  |
| Shuangliu | C11 | ovine | I | DI | **+** |  |
| Shuangliu | C12 | ovine | III | DI | - |  |
| Shuangliu | C13 | ovine | III | AS | **+** | enterovirus |
| Shuangliu | C14 | ovine | III | DI | - |  |
| Shuangliu | C15 | ovine | III | DI | - |  |
| Shuangliu | C16 | ovine | I | DI | **+** |  |
| Shuangliu | C17 | ovine | III | DI | - |  |
| Shuangliu | C18 | ovine | III | AS | - |  |
| Shuangliu | C19 | ovine | I | DI | **+** |  |
| Shuangliu | C20 | ovine | II | AS | - |  |
| Xinjin | A1 | ovine | III | AS | - |  |
| Xinjin | A2 | ovine | III | AS | - |  |
| Xinjin | A3 | ovine | III | AS | **+** | kobuvirus |
| Xinjin | A4 | ovine | III | AS | - |  |
| Xinjin | A5 | ovine | III | AS | **+** |  |
| Xinjin | A6 | ovine | III | AS | **+** |  |
| Xinjin | E1 | ovine | III | AS | - |  |
| Xinjin | E2 | ovine | III | AS | - |  |
| Xinjin | E3 | ovine | III | AS | - |  |
| Xinjin | E4 | ovine | I | DI | - | kobuvirus |
| Xinjin | E5 | ovine | III | AS | - |  |
| Xinjin | E6 | ovine | III | AS | - | kobuvirus |
| Xinjin | E7 | ovine | III | AS | - |  |
| Xinjin | E8 | ovine | III | AS | - |  |
| Xinjin | E9 | ovine | III | AS | - |  |
| Xinjin | E10 | ovine | III | AS | - | enterovirus、kobuvirus |
| Xinjin | F1 | ovine | III | AS | - |  |
| Xinjin | F2 | ovine | I | AS | - | kobuvirus |
| Xinjin | F3 | ovine | II | AS | **+** |  |
| Xinjin | F4 | ovine | III | AS | - |  |
| Xinjin | F5 | ovine | I | AS | - | enterovirus |
| Xinjin | F6 | ovine | III | AS | - |  |
| Xinjin | F7 | ovine | I | DI | **+** |  |
| Xinjin | F8 | ovine | III | AS | - |  |
| Xinjin | F9 | ovine | III | AS | - |  |
| Xinjin | F10 | ovine | III | AS | - | enterovirus |
| Xinjin | F11 | ovine | II | AS | - | enterovirus |
